# Human Herpesvirus-6 corneal Endotheliitis after intravitreal injection of Ranibizumab

**DOI:** 10.1186/s12886-019-1032-2

**Published:** 2019-01-16

**Authors:** Masahiro Onda, Yusuke Niimi, Kenji Ozawa, Ikumi Shiraki, Kiyofumi Mochizuki, Tetsuya Yamamoto, Sunao Sugita, Kyoko Ishida

**Affiliations:** 1 Department of Ophthalmology, Gifu University Graduate School of Medicine, 1-1 Yanagido, Gifu-shi, Gifu, 501-1194 Japan; 2 Laboratory for Retinal Regeneration, Riken Center for Developmental Biology, Kobe, Japan; 3 Department of Ophthalmology, Toho University Ohashi Medical Center, 2-22-36, Ohashi, Meguro-ku, Tokyo, 153-8515 Japan

**Keywords:** Human herpesvirus-6, Corneal endotheliitis, Intravitreal injection, Ranibizumab

## Abstract

**Background:**

To report the first case of human herpesvirus-6 (HHV-6) corneal endotheliitis that developed after intravitreal ranibizumab injections.

**Case presentation:**

A 63-year-old man with a medical history of diabetes and systemic steroid treatment for bullous pemphigoid had been receiving intravitreal injections of ranibizumab in the left eye for 2 years according to a Pro Re Nata treatment regimen for macular edema associated with branch retinal vein occlusion. Twenty days after the last injection, the patient presented with pain and decreased visual acuity in his left eye. His best corrected visual acuity in the left eye was 2/200, and intraocular pressure was 45 mmHg with edema of the central stromal cornea, mild conjunctival injection, intermediate keratic precipitates, and mild anterior chamber reaction. HHV-6 DNA was detected in the aqueous humor using multiplex strip polymerase chain reaction, and it was identified as variant A, HHV-6A. A diagnosis of HHV-6A-associated corneal endotheliitis was made. Oral valganciclovir and topical ganciclovir therapy was initiated with good resolution of all symptoms and signs.

**Conclusions:**

HHV-6A can be a possible complication of intravitreal ranibizumab therapy. To the best of our knowledge, this is the first reported case of HHV-6A corneal endotheliitis following intravitreal ranibizumab injection.

**Electronic supplementary material:**

The online version of this article (10.1186/s12886-019-1032-2) contains supplementary material, which is available to authorized users.

## Introduction

Human herpesvirus-6 (HHV-6) is a member of the HHV family and has been subtyped into two variants, variant A (HHV-6A) and variant B (HHV-6B) [1]. The seroprevalence of HHV-6 in the adult population approaches over 90% [2]. In the United States, certain parts of Europe, and Japan, HHV-6B is the predominant cause of childhood HHV-6 infection [3]. In these regions, primary HHV-6B infection typically occurs by the age of 2 years, and it is the etiological cause of roseola infantum, also known as exanthema subitum [3]. Like all herpesviruses, HHV-6 establishes life-long latency in human hosts, and reactivation of a latent HHV-6 infection can cause serious complications, especially in immunocompromised patients [4]. In spite of our knowledge of HHV-6B, diseases associated specifically with HHV-6A have not yet been identified, and intraocular inflammation caused by HHV-6A has been described only in a small number of cases [3, 5–9].

Intravitreal injection of anti-vascular endothelial growth factor (anti-VEGF) agents has revolutionized the treatment of common retinal disease. Their increased use, however, has led to an increase in ocular post-injection and class-associated adverse drug events [10, 11]. Although rare, serious sight-threatening complications from intravitreal injection can include elevated intraocular pressure (IOP), keratitis, uveitis, cataract, retinal detachment, and endophthalmitis.

In the present study, we describe a rare case of an immunocompromised patient who developed corneal endotheliitis after intravitreal injection with the anti-VEGF agent ranibizumab. Genomic HHV-6A DNA was detected in the aqueous humor of this patient by multiplex strip polymerase chain reaction (PCR). To our knowledge, HHV-6 corneal endotheliitis after intravitreal ranibizumab injection has not been previously reported.

## Case presentation

**Fig. 1:**
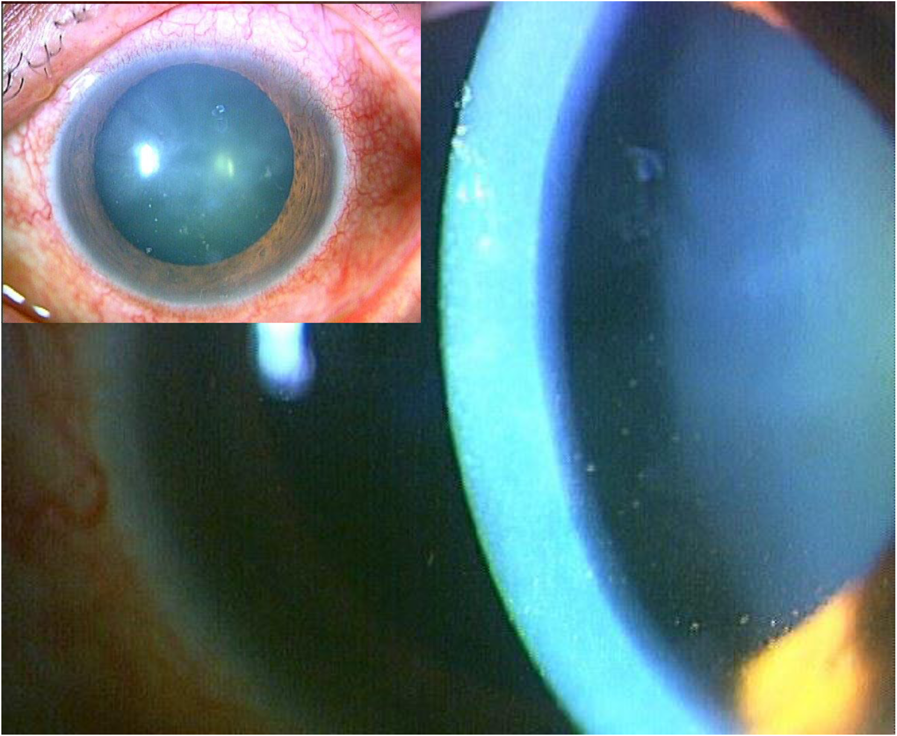
External photograph of the left eye of the patient. This photograph was taken upon initial examination showing edema of the central cornea with mild conjunctival injection (insert) and mild anterior chamber reaction with intermediate keratic precipitates

A 63-year-old Japanese man with a medical history of diabetes for 7 years, Fahr’s disease for 4 years, and systemic steroid use for bullous pemphigoid for 2 years had been receiving intravitreal injections of ranibizumab in the left eye for 2 years according to a Pro Re Nata regimen (PRN) for macular edema associated with branch retinal vein occlusion. For his diabetes, the patient had been taking an oral anti-diabetic drug, miglitol, 50 mg, 3 times daily. His steroid therapy for bullous pemphigoid had begun with prednisolone at 20 mg/day, had gradually tapered off, and continued at 5 mg/day for the past 6 months. Twenty days after the last injection, the patient presented with a 1-week duration of left eye pain. Upon examination, his best corrected visual acuity (BCVA) was 20/15 in the right eye (OD) and 20/200 in the left eye (OS), and IOP was 19 mmHg OD and 45 mmHg OS. Slit-lamp examination of the left eye revealed mild edema of the central cornea with mild conjunctival injection, intermediate keratic precipitates (KP), mild anterior chamber reaction, and incipient cataract. Coin-shaped lesions, linear KP, and iris atrophy were not present (Fig. 1). Dilated funduscopic examination of the left eye showed macular edema with hard retinal exudates secondary to a branch retinal vein occlusion. The patient’s right eye was completely normal. Endothelial cell density was 2719 cells/mm^2^ OD and 1733 cells/mm^2^ OS Additional file 1.

Laboratory tests including blood cell count, leucocytes, C-reactive protein, and angiotensin-converting enzyme were all essentially normal. Serologic tests were negative for syphilis, human immunodeficiency virus, and human T-cell leukemia virus type 1 (HTLV-1). The results of serologic testing for HHV, herpes simplex virus (HSV), varicella zoster virus (VZV), cytomegalovirus (CMV), and Epstein-Barr virus (EBV) were positive. Based on the patient’s ocular manifestations, bacterial or viral infection was suspected. Multiplex strip PCR, which can detect 24 common ocular infectious disease pathogens [12], was performed on biopsied aqueous humor. Results showed that genomic DNA of HHV-6 was present. The 23 other pathogens, e.g., HSV type 1 and 2, VZV, EBV, CMV, HHV-7, HHV-8, bacterial 16s ribosomal RNA (rRNA), and fungal 28s rRNA were however not present. The concentration of HHV-6 DNA was 3.72 × 10^3^ copies/mL, and the PCR product was specific for the HHV-6A variant sequence. Anti-HHV-6 IgG antibodies in blood samples were positive (40 times) and anti-HHV-6 IgM antibodies were negative (< 10 times).

These results led us to make the diagnosis of corneal endotheliitis with anterior uveitis related to an HHV-6 infection. We began treating the patient with 900 mg of oral valganciclovir twice daily, as well as topical 1% ganciclovir and 0.1% betamethasone 4 times per day, without discontinuation of systemic steroids. Topical anti-glaucoma agents (1% brinzolamide twice daily and 0.004% travoprost once per day) were also prescribed. Four weeks after the initiation of therapy, the copy number of HHV-6 DNA in the aqueous humor had decreased to 5.80 × 10^2^ copies/mL. Six weeks later, the corneal edema and KPs were completely resolved. Therefore, oral valganciclovir and topical anti-glaucoma agents were discontinued. Topical ganciclovir with topical steroids was continued for 8 more months. The patient’s BCVA improved to 20/80, the same level as before the episode of corneal endotheliitis, and IOP decreased to 18 mmHg OS without anti-glaucoma agents. At the patient’s one-year follow-up examination, endothelial cell density was 2786 cells/mm^2^ OD and 2545 cells/mm^2^ OS Additional file 2, and the eye was essentially normal without recurrences of corneal endotheliitis and anterior uveitis.

This study was approved by the Institutional Ethics Committee, Medical Review Board of Gifu University Graduate School of Medicine, and it adheres to the tenets of the Declaration of Helsinki. Informed consent was obtained from the patient.

## Discussion and conclusions

The use of anti-VEGF is often included in current standard care for age-related macular degeneration, diabetic retinopathy, and retinal vein occlusion. Complications from intravitreal anti-VEGF administration can be attributed to the injection procedure or drug effects [10, 11]. Activation of the virus after intravitreal anti-VEGF administration is rare, and there have been only two reports of herpetic epithelial keratitis after intravitreal bevacizumab injection [13, 14]. These reports described a 63-year-old diabetic man who developed HSV epithelial keratitis [13] and a 66-year-old diabetic man with bilateral HSV keratitis following intravitreal bevacizumab injection [14]. To the best of our knowledge, however, there have been no reports of HHV-6 ocular infection following anti-VEGF therapy. HHV-6 infection almost always occurs in children before age 2 [3], and it can latently reside in cells of the lymphoid and myeloid lineage [15–17]. Various stressors have been implicated in herpetic activation including aging, stress, trauma, surgery, and immunosuppressive diseases [13, 14]. Stressors such as these may reduce the body’s cell-mediated virus-specific immune response. Our rare case may have been due to the combination of multiple factors. Immunocompromised status due to steroid treatment of bullous pemphigoid and diabetes mellitus, the periodic stress from injections, or the injection itself may have triggered the reactivation of HHV-6.

Intraocular inflammation associated with HHV-6 infection has been reported in cases without anti-VEGF injection. Table 1 [5–9, 18–29] summarizes the clinical manifestations of these reported cases. HHV-6 may cause ocular inflammation with optic neuropathy, optic vasculitis, and uveitis (Table 1). The first reported case was of an 11-year-old girl with unilateral chronic uveitis. Diagnosis was made in 1993 by detection of anti-HHV-6 IgM and anti-HHV-6 IgG antibodies in a blood sample [18]. Oberacher-Velten et al. described a middle-aged male patient with juvenile diabetes and bilateral optic neuropathy [19]. Aside from these two cases, diagnosis was made using PCR. HHV-6 has been subtyped into two variants: variant A (HHV-6A) and variant B (HHV-6B) [1]. HHV-6A has a greater predilection for neural cells than HHV-6B [30], but it is less often associated with disease than HHV-6B is [7]. Among the reported cases of intraocular inflammation with HHV, virus subtype was identified in 6 cases as HHV-6A [5–9] and the current study] and in 9 cases as HHV-6B [7, 8, 21, 22]. In 4 of 6 cases of HHV-6A infection, however, *Toxocara canis*, CMV, and EBV were detected simultaneously [5, 7–9]. HHV-6 is closely related to CMV infection [7, 8, 27]. Yokogawa et al. found DNA of both CMV and HHV-6 in the aqueous humor of a 67-year-old man with unilateral corneal endotheliitis and a history of systemic steroid use for rheumatoid arthritis [27]. Cohen et al. tested vitreous fluid in 101 patients for HHV-6A, HHV-6B, and HHV-7 DNA by PCR, and HHV-6A DNA (4950 copies per mL) was detected in the vitreous fluid of one patient with CMV retinitis [7]. Another report described a 71-year-old woman with unilateral necrotic retinitis who had DNA of both CMV and HHV-6 in a vitreous sample [8]. Although HHV-6 reactivation frequently accompanies CMV reactivation, the presence of HHV-6A DNA in the eye may merely reflect the immunocompromised state of the patient [7]. HHV-6 can latently reside in cells of the lymphoid and myeloid lineage, and thus it is possible that it could enter the inflamed eye via immune cells, similar to EBV and HIV [8, 31–33]. Sugita et al. [8] reported 8 cases of HHV-related ocular inflammation, and in 7 of these, other infectious agents were present (*Toxocara canis*, HSV-1, CMV, and 3 cases of bacterial infection). Thus, HHV-6 DNA in circulating T cells, monocytes, and leukocytes may have been carried to the eyes via inflammatory cells as a result of destruction of the blood-retina barrier [8]. However, we were only able to detect genomic HHV-6 DNA in the aqueous humor of our patient, not DNA from the other 23 pathogens, e.g., HSV type 1 and 2, VZV, EBV, CMV, HHV-7, HHV-8, bacterial 16s ribosomal RNA (rRNA), and fungal 28s rRNA,. Furthermore, the copy number of HHV-6 DNA in the aqueous humor decreased with anti-HHV treatment. Qavi et al. found that HHV-6 was capable of infecting corneal epithelial cells in vitro, causing morphological changes similar to those caused by other human herpes viruses [34]. Significant corneal endothelial cell density changes observed in our case may have been caused by corneal endotheliitis with mild edema of the central cornea, intermediate KP, and mild anterior chamber reaction. Thus, HHV-6 could be one of the causative agents of corneal endotheliitis.

**Table 1:** Summary of reported cases of intraocular inflammation associated with HHV-6 infection

Author, year [reference umber]	Age (years)	Gender	Eye	DM	Other associated diseases	Ocular disease and complications	HHV-6 detection		Variant	Treatment	Clinical outcomes
							aqueous humor	blood			
Wiersbitzky, 1993 [18]	11	F	OS	–	None	Chronic iridocyclitis	NA	Anti-HHV-6 ab		NA	NA
Oberacher-Velten, 2005 [19]	31	M	OU	+	–	Optic neuropathy, unilateral tonic pupil	NA	Positive HHV-6 titers		Foscarnet, methylprednisolone	Optic nerve atrophy. Reaction of the pupil became normal. VA improved to 20/25 OD and 20/20 OS
Takizawa, 2006 [20]	1	F	OS	–	–	Central retinal vein occlusion by optic disc vasculitis	NA	+ HHV-6 DNA by PCR		Intravenous methylprednisolone	Optic disc atrophy, fundus atrophy. Unmeasurable VA
Moschettini, 2006 [21]	23	F	OU	–	None	Recurrent anterior optic neuritis and granulomatous iridocyclitis	NA	+ (also in CSF) by PCR	HHV-6B	Intravenous and oral glucocorticoids, Ganciclovir	Patient had optic neuritis 4 times. After last relapse, patient was successfully treated with ganciclovir.
Méchaï, 2007 [22]	59	F	OU	–	HIV	Retrobulbar optic neuritis and meningoencephalitis	NA	+ (also in CSF) by PCR	HHV-6B	Foscarnet and ganciclovir, then changed to cidofovir and long-term valganciclovir	By 12 months, the optic neuritis had almost resolved fully. VA was 20/20 (OU).
Sugita, 2007 [5]	75	M	OD	NA	Anti-*Toxocara canis* IgG in serum, aqueous humor, and vitreous	Severe panuveitis	+ (in also vitreous) by PCR	+ by PCR, Positive HHV-6 titer	HHV-6A	Diagnostic vitrectomy, valganciclovir, systemic corticosteroids	Resolution of vitreous opacities, disc swelling, and retinal exudates. HHV-6A DNA levels decreased in aqueous humor.
Maslin, 2007 [6]	81	M	OU	+	AML (in remission after chemotherapy)	Acute uveitis, disc edema Meningitis	+ by PCR	+ (in CSF) by PCR, Positive HHV-6 titer	HHV-6A	Foscarnet, ganciclovir	Disc edema was totally regressed. VA improved to the previous level of 20/40 (OD) and 20/32 (OS). HHV-6 was negative in aqueous humor and cerebrospinal fluid on day 45.
Cohen, 2010 [7]	NA	NA	NA	NA	Case 1; CMV retinitis	Case 1: Ocular inflammation	+ (in vitreous) by PCR Of 101 samples, Case 1 was positive for HHV-6A and CMV	NA	Case 1: HHV-6A	NA	NA
	NA	NA	NA	NA	Case 2; none	Case 2: Ocular inflammation	+ (in vitreous) by PCR	NA	Case 2: HHV-6B	NA	NA
Groot-mijines, 2010 [23]	42	M	OD	–	–	Uveitis with heterochromia	+ (in vitreous) by PCR	NA		Diagnostic vitrectomy	Patient regained full VA
Glâtre F, 2010 [24]	47	F	Unilateral	–	RA, TB, Toxicoplasmic co-infection	panuveitis	+ by PCR	NA		Ganciclovir for HHV-6, and pyrimethamine and sulfadiazine for Toxicoplasma	NA
Ogata, 2011 [25]	63	M	OS	–	none	Uveitis with optic neuritis	+by PCR	Positive HHV-6 titer		Systemic corticosteroids, aspirin	VA improved to 10/20
Okuno T, 2011 [26]	N/A	N/A	NA	NA	HSV-1 was detected in 4 cases	Corneal ulcer in 6 cases and keratouveitis in 3 cases	+(in smear samples from corneal ulcer or keratouveitis) by PCR	NA		Acyclovir	NA
Sugita 2012 [8]	64	F	OS	NA	HSV-1	Corneal endotheliitis	+by PCR	N/A	HHV-6B	Valganciclovir	NA
	70	M	OS	NA	bacteria (+)	Endophthalmitis, retinal necrosis	+ (in vitreous) by PCR	N/A	HHV-6B	Vitrectomy, antibiotics	NA
	74	F	OD	NA	*P. acnes*	Postoperative endotheliitis	+(in vitreous) by PCR	N/A	HHV-6B	Vitrectomy, valganciclovir	NA
	79	F	OS	NA	bacteria (+)	Postoperative endotheliitis	+(in vitreous) by PCR	N/A	HHV-6B	Vitrectomy, steroids, antibiotics	NA
	71	F	OS	NA	CMV	Necrotic retinitis	+(in vitreous) by PCR	N/A	HHV-6A	Valganciclovir	NA
	24	F	OS	NA	none	uveitis	+by PCR	N/A	HHV-6B	none	NA
	22	M	OD	NA	bacteria (+)	keratitis	+(in cornea) by PCR	N/A	HHV-6B	Antibiotics	NA
Yokogawa, 2013 [27]	67	M	OD	–	RA CMV	Corneal endotheliitis	+ (Both HHV-6 and CMV) by PCR	NA		Valganciclovir, topical 0.5% ganciclovir 0.1% betamethasone	Corneal endotheliitis resolved VA improved to 20/25
Malamos, 2013 [28]	64	M	OU	–	HIV	Posterior uveitis	+ (in vitreous) by PCR	+ Positive HHV-6 titer		Diagnostic vitrectomy, valganciclovir	Complete remission of inflammation
Bajric, 2014 [29]	59	M	OU	–	Multiple myeloma	Panuveitis, encephalitis	Negative by PCR	+ (in CSF) by PCR	+ by PCR	Ganciclovir, foscarnet, intravitreal foscarnet	Encephalitis resolved. VA improved to 20/20 (OD) and 20/30 (OS) with complete resolution of retinal hemorrhages and no evidence of active uveitis.
Papageorgious, 2014 [9]	22	M	OU	–	EBV	Acute retinal necrosis, fourth nerve palsy, encephalitis	Not performed	+ (in CSF) by PCR	HHV-6A	Acyclovir, valacyclovir, methylprednisolone	VA improved to 20/20 (OU). There was no residual visual field defect, and binocular diplopia improved.
Current report, 2018	63	M	OS	+	Fahr’s disease, bullous pemphigoid	Corneal endotheliitis	+ by PCR	+ by PCR	HHV-6A	Oral valganciclovir, topical ganciclovir	VA improved to 20/80, the same level before the episode of corneal endotheliitis.

*HHV-6* Human herpesvirus-6, *DM* Diabetes mellitus, *NA* Information not available, *VA* Visual acuity, *OD* Oculus dexter, *OS* Oculus sinister, *PCR* Polymerase chain reaction, *CSF* Cerebrospinal fluid, *HHV-6B* Human herpesvirus-6 variant B, *OU* Oculus uterque, *HHV-6A* Human herpesvirus-6 variant A, *ALM* Acute type 1 myeloid leukemia, *CMV* Cytomegalovirus, *RA* Rheumatoid arthritis, *TB* Tuberculosis, *HSV-1* Human T-cell leukemia virus type 1, *HIV* Human Immunodeficiency Virus, *EBV* Epstein-Barr virus

Aside from the cases of other suspicious ocular infections, HHV-6 alone was detected in 11 cases: HHV-6A in 2 cases [6] and the current study, HHV-6B in 3 cases [7, 8, 21], and HHV-6 in 6 cases [18–20, 23, 25, 29]. Among these cases, 4 were immunocompromised patients, 5 were immunocompetent, and detailed information from one patient was not available. Four of these 10 cases, from which patient information was available, were females, and 6 were males. Six cases were unilateral, and 4 were bilateral. Patients’ ages ranged from 1 to 81 years, and the average age was 39.8. It is possible that differences in clinical presentation can be related to infection by the different viral variations [29]. With HHV-6A intraocular infection, only 2 cases (our case and Maslin’s case [6]) reported infection of HHV-6A DNA with no other pathogens present. Both Maslin’s case [6] and ours were elderly men with immunocompromised statuses.

Of the 13 cases [5, 6, 9, 19–23, 25, 27–29], and the current study which reported on prognosis, 11 (85%) saw improvements following treatment (10 cases had anti-viral therapy; 1 had vitrectomy). A 1-year-old girl manifesting unilateral central retinal vein occlusion in one eye with exanthema subitum later experienced optic disc and fundus atrophy with unmeasurable BCVA [20]. A case of uveitis with optic neuritis in a healthy, elderly male patient resulted in a measured BCVA of 10/20 [25]. These two cases were treated with systemic steroids, but not antiviral drugs. There are currently no FDA-approval antiviral drugs for the treatment of HHV-6 infection [35]. However, foscarnet, ganciclovir, and cidofovir have shown efficacy against HHV-6 in vitro [36] and are currently used to treat HHV-6-related intraocular inflammation in clinical settings (Table 1).

In conclusion, we report the first case of HHV-6A corneal endotheliitis after intravitreal ranibizumab injection. HHV-6A endotheliitis can be a possible complication of intravitreal ranibizumab therapy. This may present as localized corneal edema with KPs, IOP elevation, and corneal endothelial damage. To confirm the diagnosis, multiplex strip PCR using ocular fluid can be used. The use of topical and/or systemic valganciclovir and ganciclovir is effective.

## Data sharing

Data sharing is not applicable to this article as no datasets were generated during case report.

## Additional files

Additional file 1: Corneal specular microscopy OS at the onset. The endothelium image was obtained by Konan Non–Con Robo Specular Microscope (Konan Medical, Inc., Hyogo, Japan). CD: endotheliam cell density, SD: standard deviation, CV: coefficient of value, 6A: hexagonal cell ratio, Pachy: central corneal thickness. Endothelial cell density was 1733 cells/mm^2^. Corneal pachymetry of 597 μm indicates mild edema. (JPG 72 kb)

Additional file 2: Corneal specular microscopy OS at 1-year follow-up. The endothelium image shows that endothelial cell density and corneal thickness are normal. (JPG 82 kb)

## Abbreviations

anti-VEGF: Anti-vascular endothelial growth factor
BCVA: Best corrected visual acuity
CMV: Cytomegalovirus
EBV: Epstein-Barr virus
HHV-6: Human herpesvirus-6
HHV-6A: Human herpesvirus-6 variant A
HHV-6B: Human herpesvirus-6 variant B
HIV: Human immunodeficiency virus
HSV: Herpes simplex virus
HTLV-1: Human T-cell leukemia virus type 1
IOP: Intraocular pressure
KP: Keratic precipitates
OD: Oculus dexter
OS: Oculus sinister
PCR: Polymerase chain reaction
PRN: Pro Re Nata regimen
rRNA: Ribosomal RNA
VZV: Varicella zoster virus
