# Supplementary figures and images for: Human Herpesvirus-6 corneal Endotheliitis after intravitreal injection of Ranibizumab
Source: BMC Ophthalmol. 2019 Jan 16;19:19. doi: 10.1186/s12886-019-1032-2 (PMC6335734; doi:10.1186/s12886-019-1032-2)

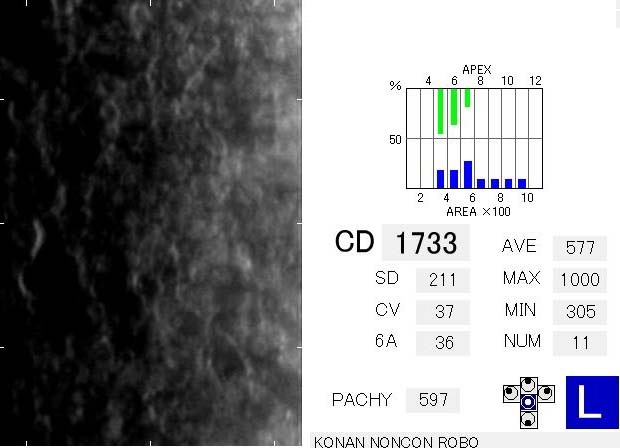

Supplement: Supplementary file 1 — Corneal specular microscopy OS at the onset. The endothelium image was obtained by Konan Non–Con Robo Specular Microscope (Konan Medical, Inc., Hyogo, Japan). CD: endotheliam cell density, SD: standard deviation, CV: coefficient of value, 6A: hexagonal cell ratio, Pachy: central corneal thickness. Endothelial cell density was 1733 cells/mm2. Corneal pachymetry of 597 μm indicates mild edema. (JPG 72 kb) [file 12886_2019_1032_MOESM1_ESM.jpg]

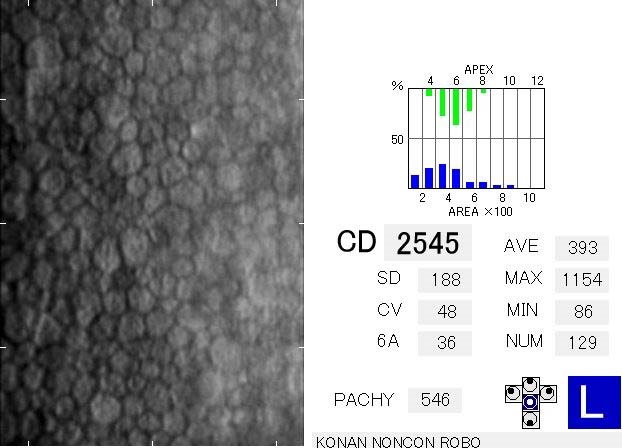

Supplement: Supplementary file 2 — Corneal specular microscopy OS at 1-year follow-up. The endothelium image shows that endothelial cell density and corneal thickness are normal. (JPG 82 kb) [file 12886_2019_1032_MOESM2_ESM.jpg]
